# Inhibition of fatty acid oxidation modulates immunosuppressive functions of myeloid-derived suppressor cells and enhances cancer therapies

**DOI:** 10.1186/2051-1426-3-S2-O18

**Published:** 2015-11-04

**Authors:** Amir A Al-Khami, Fokhrul Hossain, Dorota Wyczechowska, Claudia Hernandez, Liqin Zheng, Krzystoff Reiss, Luis Del Valle, Jimena Trillo-Tinoco, Tomasz Maj, Weiping Zou, Paulo C Rodriguez, Augusto C Ochoa

**Affiliations:** 1 Stanley S. Scott Cancer Center, Louisiana State University Health Sciences Center, New Orleans, LA, USA; 2 Department of Surgery, University of Michigan, Ann Arbor, MI, USA

## 

Myeloid-derived suppressor cells (MDSC) promote tumor growth by inhibiting T cell immunity and supporting malignant cell proliferation and migration. The therapeutic potential of blocking MDSC in tumors has been limited by their heterogeneity, plasticity, and resistance to various chemotherapy agents. Recent studies have highlighted the role of energy metabolic pathways in the differentiation and function of immune cells; however, the metabolic characteristics regulating MDSC remain unclear. We aimed to determine the energy metabolic pathway(s) used by MDSC, establish its impact on MDSC function, and test whether its inhibition blocks MDSC and enhances anti-tumor therapies. Using several murine tumor models, we found that tumor-infiltrating MDSC (T-MDSC) increased fatty acid uptake and activated fatty acid oxidation (FAO). This was accompanied by an increased mitochondrial mass, upregulation of key FAO enzymes, and increased oxygen consumption rate. Pharmacological inhibition of FAO blocked immune inhibitory pathways and functions in T-MDSC and decreased their production of inhibitory cytokines. FAO inhibition alone significantly delayed tumor growth in a T cell-dependent manner and enhanced the anti-tumor effect of adoptive T cell therapy. Furthermore, FAO inhibition combined with low-dose chemotherapy completely inhibited T-MDSC immunosuppressive effects and induced a significant anti-tumor effect. Interestingly, a similar increase in fatty acid uptake and expression of FAO-related enzymes was found in human MDSC in peripheral blood and tumors. These results support the possibility of testing FAO inhibition as a novel approach to block MDSC and enhance various cancer therapies.

## Consent

Written informed consent was obtained from the patient for publication of this abstract and any accompanying images. A copy of the written consent is available for review by the Editor of this journal.

